# Intraperitoneal 8-OH-DPAT reduces competition from contextual but not discrete conditioning cues

**DOI:** 10.1016/j.pbb.2019.172797

**Published:** 2019-12

**Authors:** H.J. Cassaday, K.E. Thur

**Affiliations:** School of Psychology, University of Nottingham, Nottingham, UK

**Keywords:** Fear conditioning, Trace conditioning, Contextual conditioning, Overshadowing, 5-Hydroxytyptamine, Rat

## Abstract

The effects of the serotonergic (5-hydroxytryptamine, 5-HT) agonist 8-hydroxy-2-(di-n-propylamino)tetralin (8-OH-DPAT; 0.2 and 0.4 mg/kg i.p.) were examined in trace conditioning (Experiment 1) and overshadowing (Experiment 2) procedures. Both experiments used a fear conditioning procedure conducted off-the-baseline in water deprived male Wistar rats. 8-OH-DPAT was administered during conditioning and its effects were examined drug free as the suppression of an established licking response, both upon re-exposure to the cues provided by the conditioning chambers and upon presentation of experimental stimuli. There were no statistically significant effects of 8-OH-DPAT on conditioning to the discrete cue provided by a 5 s conditioned stimulus (CS), irrespective of the length of the trace interval used in Experiment 1, and irrespective of whether the CS took the form of a light alone, or a noise plus light compound in the Experiment 2 overshadowing procedure. The successful demonstration of overshadowing required the use of a second conditioning session which allowed further evaluation of the effects of 8-OH-DPAT in that neither a weak nor a strong overshadowing effect was modulated by either drug dose. Nonetheless conditioning to contextual cues was attenuated by treatment with 8-OH-DPAT at the 30 s trace interval. We therefore conclude that 8-OH-DPAT reduces competition from contextual but not discrete conditioning cues. This pattern of results lends further support to the view that contextual cue conditioning and discrete cue conditioning are modulated by different neuropharmacological mechanisms.

## Highlights

• 8-OH-DPAT (0.2 and 0.4 mg/kg i.p.) was tested in two fear conditioning procedures.
• 8-OH-DPAT reduced conditioning to contextual cues at a 30 s trace.
• However, overshadowing produced by presentation of a compound cue was unaffected by 8-OH-DPAT.
• 8-OH-DPAT reduced competition from contextual but not discrete conditioning cues.

## 1 Introduction

The serotonergic (5-hydroxytryptamine, 5-HT) system is involved in a variety of cognitive and behavioural processes, including various aspects of learning and memory (Altman and Normile, 1988; McEntee and Crook, 1991; Meneses and Perez-Garcia, 2007). Amongst the multiplicity of 5-HT receptor sub-types, the 5-HT_1A_ site in particular has been implicated in learning and memory (Cassaday and Gaffan, 1996; Cassaday et al., 2000; Manuel-Apolinar and Meneses, 2004; Eriksson et al., 2008; Costa et al., 2012). Perhaps surprisingly, except under restricted conditions (e.g., Avanzi et al., 2003), fear conditioning with discrete experimental cues has been reported to be unaffected by treatment with 5-HT_1A_ agonists (Inoue et al., 2011). However, consistent with such compounds' potential efficacy as anxiolytics, at least in pre-clinical tests (Cheeta et al., 2001; Borsini et al., 2002), contextual fear conditioning is reliably reduced (Li et al., 2001, Li et al., 2006), in some cases together with discrete cue conditioning (Youn et al., 2009).

Simple associative learning as exemplified in such fear conditioning procedures is a fundamental process, essential to a range of higher order cognitive processes, which lends itself to a variety of procedural variants to manipulate the reliability with which the discrete cue or conditioned stimulus (CS, e.g. light or noise) predicts a motivationally significant outcome (unconditioned stimulus, UCS, e.g. foot shock). Trace conditioning procedures use the introduction of a time interval to manipulate CS associability: normally a CS closely followed by the UCS becomes better associated than does a CS followed by a longer intervening interval to the UCS (Kamin, 1965). Overshadowing may contribute to the difficulty in conditioning over a trace interval in that intervening contextual cues typically acquire associative strength. Moreover, the trace conditioning procedure has been adapted to present an experimental background stimulus which provides an additional measure of contextual conditioning (Rawlins and Tanner, 1998; Norman and Cassaday, 2003; Horsley and Cassaday, 2007; Thur et al., 2014). Contextual conditioning can also be assessed by measuring animals' subsequent responses to the experimental chambers in which they were conditioned (Odling-Smee, 1975; Rescorla and Wagner, 1972). Irrespective of the role of contextual cues, the ability to bridge temporal intervals has been argued to provide a measure of working memory function (Sweatt, 2004; Grimond-Billa et al., 2008). Overshadowing procedures use the relative intensity of competing CSs to manipulate associability: normally a relatively more intense CS acquires associative strength at the expense of a relatively less intense CS (Pavlov, 1927).

In the present study, both trace conditioning (Experiment 1) and overshadowing (Experiment 2) were examined using a fear conditioning procedure (suppression of drinking after conditioning with foot shock UCS) that we have previously used to test the effects of indirect dopamine (DA) agonists (Norman and Cassaday, 2003; Horsley and Cassaday, 2007; Nelson et al., 2011). Hyper-dopaminergic animals both condition more than normally over an extended trace interval and show increased suppression to contextual cues (Norman and Cassaday, 2003; Horsley and Cassaday, 2007). Opponent interactions between DA and 5-HT have long been documented (Lucki and Harvey, 1979), but although the close interplay between these systems is indisputable, the nature of the interaction can vary depending on the receptor sub-types (Alex and Pehek, 2007) and (relatedly) the brain regions examined (e.g. in NAc shell: Nelson et al., 2011, Nelson et al., 2012).

To examine the role of 5-HT_1A_ receptors in trace conditioning and overshadowing, we used 8-hydroxy-2-(di-n-propylamino)tetralin (8-OH-DPAT; 0.2 and 0.4 mg/kg i.p.). Aspects of the cognitively enhancing profile of 8-OH-DPAT are now attributed to 5-HT_7_ receptor occupancy (Meneses and Terrón, 2001). However, as a full agonist at both pre-and postsynaptic 5-HT_1A_ receptors, 8-OH-DPAT is still commonly used as a benchmark compound (e.g., Li et al., 2006; Eriksson et al., 2008; Youn et al., 2009; Zeeb et al., 2009; Costa et al., 2012). In both experiments, there were behavioural controls for any generally anxiolytic effects and predictions are based on the evidence that 5-HT_1A_ agonists are generally cognitively enhancing. Treatment with 8-OH-DPAT was predicted to increase conditioning over an extended trace interval in Experiment 1 because of improved working memory function (Sweatt, 2004; Grimond-Billa et al., 2008). In line with earlier evidence, contextual fear conditioning was predicted to be reduced (Li et al., 2001, Li et al., 2006; Inoue et al., 2011; Homberg, 2012). Reduced contextual conditioning is consistent with increased overshadowing by the available discrete cues. 8-OH-DPAT effects on overshadowing were further examined in Experiment 2.

## 2 Methods and methods

### 2.1 Animals

For each experiment, 72 experimentally naïve adult male Wistar rats (Charles River, UK) were caged in pairs on a 12:12 h light/dark cycle with food and water ad libitum. They were handled for approximately 5 min per day for 1 week and then placed on water deprivation 24 h prior to behavioural procedures which were run in the following week. In Experiment 1, the mean start weight was 220 g (range 195–243 g). In Experiment 2, the mean start weight was 219 g (range 196–238 g). Allocation to experimental groups was counterbalanced by cage rather than truly randomised.

All procedures were carried out in accordance with the United Kingdom (UK) Animals Scientific Procedures Act 1986, Project Licence number: PPL 40/3163.

### 2.2 Drugs

8-OH-DPAT HBr (Tocris, UK) was dissolved in saline at 0.2 and 0.4 mg/ml (calculated as free base) for injection (i.p.) at 1 ml/kg to administer a dose of 0.2 or 0.4 mg/kg. Control rats were injected with the equivalent volume of saline. Drug or control injections were administered 15 min prior to the conditioning stage of the procedure.

### 2.3 Behavioural conditioning apparatus

Six identical fully automated conditioning boxes, housed within sound-attenuating cases containing ventilation fans (Cambridge Cognition, Cambridge, UK), were used. The inner conditioning box walls consisted of plain steel (25 cm × 25 cm × 22 cm high) with a Plexiglas door (27 cm × 21 cm high) at the front. The floor was a shock grid with steel bars 1 cm apart and 1 cm above the lip of a 7 cm deep sawdust tray. A waterspout was mounted on one wall. The spout was 5 cm above the floor and connected to a lickometer supplied by a pump. Licks were registered by a break in the photo beam within the spout, which also triggered water delivery of 0.05 ml per lick. The waterspout was illuminated when water was available. A loudspeaker for the presentation of auditory stimuli was set in the roof. Flashing light stimuli were provided by the three wall-mounted stimulus lights and the house light flashing both on (0.5 s) and off (0.5 s).

In Experiment 1, a 5 s mixed frequency noise set at 85 dB served as the CS, presented with 3 s or 30 s trace interval to the UCS, and continuous flashing lights provided an experimental background stimulus in both trace groups. The house light was otherwise on (outside of the conditioning sessions and the subsequent test presentations of the flashing light background). In Experiment 2, a flashing light of overall 5 s duration served as the CS for the control group of rats. In the overshadowed groups, the 5 s light CS was presented in compound with a 5 s mixed frequency noise set at 85 dB. The house light was otherwise on (outside of the conditioning and test presentations of the flashing light CS).

In both experiments, foot shock of 1 s duration and 1 mA intensity provided the UCS. This was delivered through the grid floor by a constant current shock generator (pulsed voltage: output square wave 10 ms on, 80 ms off, 370 V peak under no load conditions, MISAC Systems, Newbury, UK).

Stimulus control and data collection was by an Acorn Archimedes RISC computer programmed in Basic with additional interfacing using an Arachnid extension (Cambridge Cognition).

### 2.4 Behavioural conditioning procedures

Behavioural procedures adopted parameters earlier found to reliably demonstrate reduced conditioning due the introduction of a 30 s trace interval (Norman and Cassaday, 2003; Grimond-Billa et al., 2008; Thur et al., 2014) or an explicitly competing cue in overshadowing (Nelson et al., 2011; Cassaday and Thur, 2015) using the same fear conditioning procedure. Water deprivation was introduced 1 day prior to shaping. Thereafter, the animals received 1 h and 15 min of ad libitum access to water in their home cage in addition to water in the experimental boxes. The stages of the behavioural procedure were all fully automated as detailed below.

*Pre-conditioning to establish baseline lick response*: In order to initiate licking behaviour, rats were placed in the conditioning boxes with their respective cage mate and were shaped for 1 day until all drank from the waterspout. No data were recorded. Thereafter, animals were individually assigned to a conditioning box for the duration of the experiment (counterbalanced by experimental group).
  There then followed 5 days of pre-training, in which rats drank in their conditioning boxes for 15 min each day (timed from first lick). The drinking spout was illuminated throughout, but no other stimuli were presented in this phase. Latency to first lick was recorded to assess any pre-existing differences in readiness to drink (prior to conditioning).
*Conditioning with foot shock*: Conditioning was conducted following pre-training. No water was available within the box and the waterspout was not illuminated. There were 2 conditioning trials in which the UCS foot shock was delivered following the trace interval (in Experiment 1) or the CS offset (in Experiment 2). The first pairing of CS and UCS was presented after 5 min had elapsed, and the second pairing was 5 min after the first, followed by a further 5 min left in the apparatus. In the absence of drinking, there were no behavioural measures to record.
  In the Experiment 1 trace conditioning procedure, the UCS foot shock was delivered with either a 3 or 30 s trace interval following termination of the noise CS. The flashing light background stimulus was presented for the full conditioning session duration (of 15 min plus the trace intervals). In the Experiment 2 overshadowing procedure, the CS was provided by the flashing light stimulus, either on its own in the control group or compounded with the noise stimulus, and the UCS foot shock followed immediately upon the CS offset.
*Reshaping after foot shock*: On the day following conditioning, animals were reshaped, following the same procedure as in the pre-conditioning sessions. This was in order to re-establish drinking after conditioning and provided a measure of contextual conditioning, as reflected in the extent to which drinking was suppressed in the experimental chambers on the day following conditioning.
*Conditioned suppression tests*: On the day following reshaping, the animals were placed in the conditioning boxes and presented with the CS. Water was available throughout the test and the waterspout was illuminated. The test sessions were initiated box-by-box, triggered individually by the first lick. Once the animals had made in total 50 licks, the CS was presented for 15 min. The latency to make 50 licks in the absence of the CS (the A period, timed following the first lick made in each box) provided a measure of any individual variation in baseline lick responding. This was compared with the time taken to complete 50 licks following CS onset (B period) in a suppression ratio (A / (A + B)) to assess the level of conditioning to the CS, adjusted for any individual variation in drink rate.

There were two test presentations conducted 24 h apart, thus 24 and 48 h after reshaping. In Experiment 1 the noise CS was tested first and the background light test was conducted second for all animals (Thur et al., 2014). In common with other overshadowing studies which we have reported, in Experiment 2 the overshadowed (light) stimulus was tested first and the overshadowing (noise) stimulus was tested second (Nelson et al., 2011; Cassaday and Thur, 2015).

In Experiment 2 (only), following completion of the above, animals underwent 1 baseline day to re-establish drinking. There then followed the same behavioural procedure as before: conditioning, reshaping and two further test sessions. The same drug administration (saline, 0.2 or 0.4 mg/kg 8-OH-DPAT, 15 min prior) was repeated at the conditioning stage.

### 2.5 Experimental design and analysis

In both experiments, there were 6 experimental groups run in a 3 × 2 independent factorial designs (N = 12/group) with drug, at levels saline, 0.2 mg/kg, and 0.4 mg/kg, and conditioning group, at levels 3 s and 30 s trace interval in Experiment 1, and control and overshadowed in Experiment 2. The same design was applied to analyses of variance (ANOVA) for the pre-conditioning baselines, to check for pre-existing differences by experimental condition-to-be, the reshaping latencies, to examine differences in contextual conditioning, suppression to the CS, suppression to the experimental background stimulus (in Experiment 1), and suppression to the competing tone stimulus (in Experiment 2). ANOVA of the pre-conditioning latencies included the additional factor of days (at 5 levels). *t*-Tests (two-tailed) were used to explore significant interactions. In each case alpha was set at p < 0.05 for the rejection of the null hypothesis. The dependent variables were the initial lick latencies (time to first lick) at pre-conditioning and reshaping, and the A periods and suppression ratios for the conditioning tests. Where necessary (for time to first lick at reshape) raw latency data were log transformed so that their distribution was suitable for parametric analysis.

## 3 Results

### 3.1 Experiment 1: effects of 8-OH-DPAT in a trace conditioning procedure

#### 3.1.1 Pre-conditioning - baseline lick latencies

Latencies to drink declined over the 5 days of baseline training, reflected in a main effect of days [*F*(4,264) = 23.805, p < 0.001]. However, there was no overall effect of drug or behavioural condition-to-be, nor any interaction between these factors [maximum *F*(2,66) = 0.542].

#### 3.1.2 Reshaping - conditioning effects on lick latencies

**Fig. 1:**
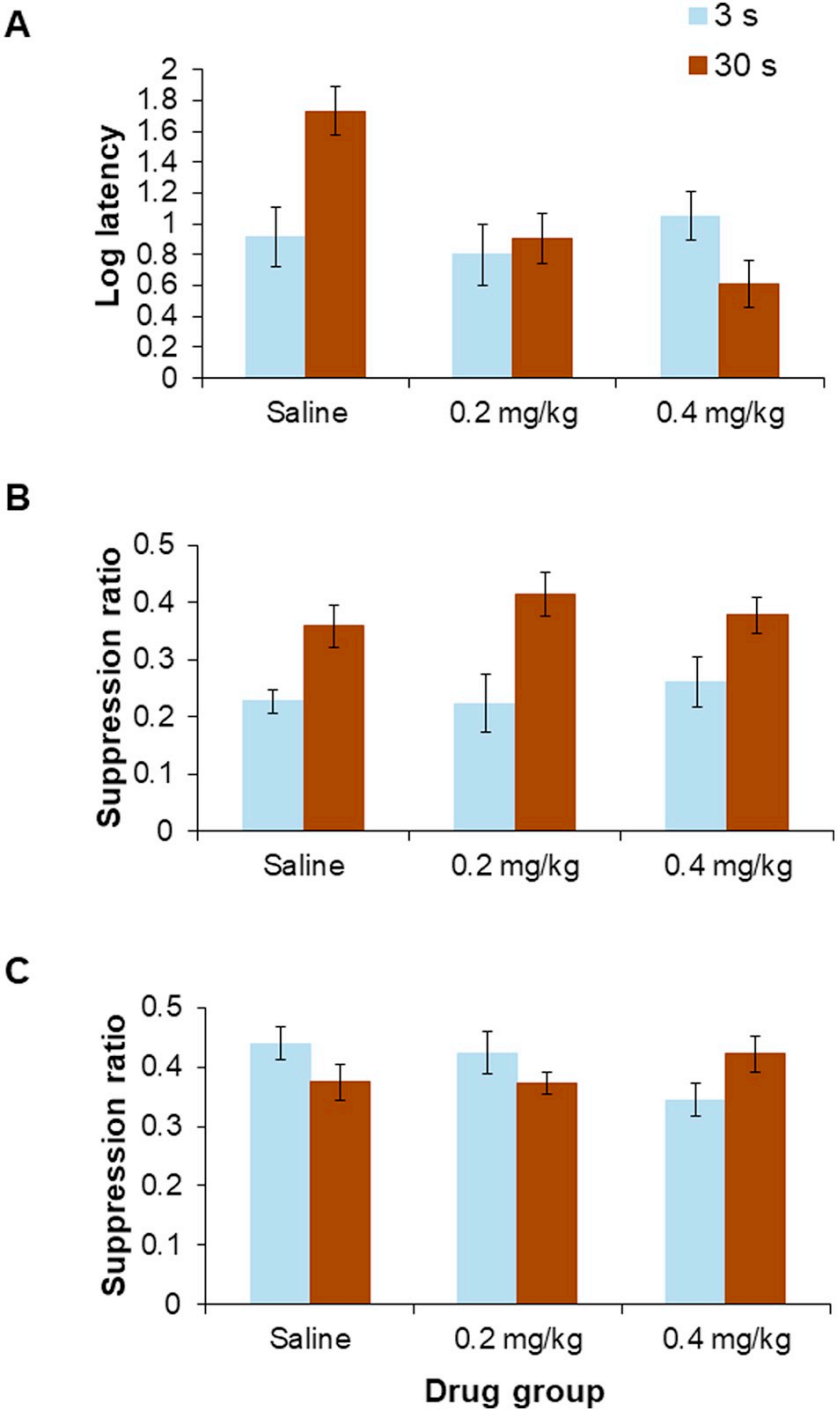
Experiment 1 (A) mean latency (log s) to drink in the experimental context at the reshape session of Experiment 1 (±S.E.M.), for rats previously conditioned in different drug groups (saline, 0.2 or 0.4 mg/kg 8-OH-DPAT) at a 3 s (light blue bars) or 30 s (dark red bars) trace interval; (B) mean suppression ratio (±S.E.M.) to the noise conditioned stimulus for rats previously conditioned in different drug groups (saline, 0.2 or 0.4 mg/kg 8-OH-DPAT) at a 3 s (light blue bars) or 30 s (dark red bars) trace intervals; (C) mean suppression ratio (±S.E.M.) to the light background stimulus for rats previously conditioned in different drug groups (saline, 0.2 or 0.4 mg/kg 8-OH-DPAT) at a 3 s (light blue bars) or 30 s (dark red bars) trace intervals.

There was no main effect of trace conditioning group on latency to drink in the reshape stage [*F*(1,66) = 1.319]. However, there was a main effect of drug [*F*(2,66) = 5.377, p < 0.01] reflecting overall shorter latencies in animals treated with 8-OH-DPAT. Moreover, there was a drug by group interaction [*F*(2,66) = 6.917, p < 0.005]. Fig. 1A shows that the 30 s trace conditioned rats treated with saline took longer to drink than their counterparts conditioned with a 3 s trace interval, whereas this pattern was reversed in the 30 s trace conditioned rats treated with 0.4 mg/kg 8-OH-DPAT, which took less time to drink than their counterparts conditioned with a 3 s trace interval. The difference between 3 and 30 s conditioned groups was significant under saline [*t*(22) = 3.292, p < 0.005], and the *reversed* pattern of difference under 0.4 mg/kg 8-OH-DPAT was marginally significant [*t*(22) = 2.028, p = 0.055].

#### 3.1.3 Conditioned suppression tests

Prior to presentation of the noise CS, rats took the same amount of time overall to make 50 licks during the A period, by both drug and trace condition [maximum *F*(1,66) = 1.350]. On the suppression ratio measure of learning, there was a significant effect of conditioning group [*F*(1,66) = 21.935, p < 0.001], reflecting an overall reduction in suppression in the 30 s trace group (see Fig. 1B). There was no effect of drug, and no drug by group interaction [maximum *F*(2,66) = 0.525].

In the Experiment 1 trace conditioning procedure, suppression to test presentations of the light background stimulus provided an additional index of contextual conditioning, in this case measured in the same way as discrete cue conditioning, by suppression ratios. Statistically, there was no main effect of drug or trace group [maximum *F*(1,66) = 0.341], but there was a significant trace by drug interaction [*F*(2,66) = 3.625, p < 0.05]. Fig. 1C shows that at the 30 s trace, both saline and 0.2 mg/kg 8-OH-DPAT groups were relatively more suppressed to the light background than the corresponding 3 s trace conditioned group, whereas this direction of effects was reversed in the trace groups treated with 0.4 mg/kg 8-OH-DPAT. Whilst this pattern of effects corresponds to that seen for contextual conditioning measured by the reshape latencies, for the measure of contextual conditioning provided by suppression to the experimental background stimulus, the differences by trace interval were not significant for any of the three drug groups [maximum *t*(22) = 1.894, p = 0.072, for the ‘reversal’ seen under 0.4 mg/kg 8-OH-DPAT].

### 3.2 Experiment 2: effects of 8-OH-DPAT in an overshadowing procedure

#### 3.2.1 Pre-conditioning - baseline lick latencies

As expected, animals drank more readily on successive days as they habituated to the experimental chambers and this was reflected statistically in a main effect of days [*F*(4,264) = 15.673, p < 0.001]. Prior to conditioning, there were no overall differences in latency to drink in the experimental chambers as a function of drug or conditioning group-to-be, neither was there any interaction [maximum *F*(2,66) = 1.571].

#### 3.2.2 Reshaping - conditioning effects on lick latencies

There was a main effect of conditioning group on latency to drink at both the first [*F*(1,66) = 14.643, p < 0.001] and second reshape session [*F*(1,66) = 6.336, p < 0.05]. Control rats conditioned to the light alone took an overall longer time to drink than overshadowed rats (Table 1). In neither test session was there any effect of drug or drug x group interaction on latency to drink at reshape [maximum *F*(2,66) = 1.890].

**Table 1:** Mean latency (log s) to drink in the experimental context at the reshape session of Experiment 2 (±S.E.M.) subsequent to (A) one or (B) two conditioning sessions for groups control (Light CS) and compound conditioned (Light + Noise CS) following in total one (A) or two (B) treatments with saline, 0.2 or 0.4 mg/kg 8-OH-DPAT.

Drug/conditioning group	Saline	0.2 mg/kg 8-OH-DPAT	0.4 mg/kg 8-OH-DPAT
(A): Light CS	1.248 (±0.185)	1.141 (±0.195)	1.039 (±0.155)
(A): Light + Noise CS	0.425 (±0.102)	0.659(±0.168)	0.839 (±0.140)
(B): Light CS	0.988 (±0.147)	1.056 (±0.178)	1.213 (±0.211)
(B): Light + Noise CS	0.505 (±0.184)	0.955 (±0.131)	0.721 (±0.185)

#### 3.2.3 Conditioned suppression tests

**Fig. 2:**
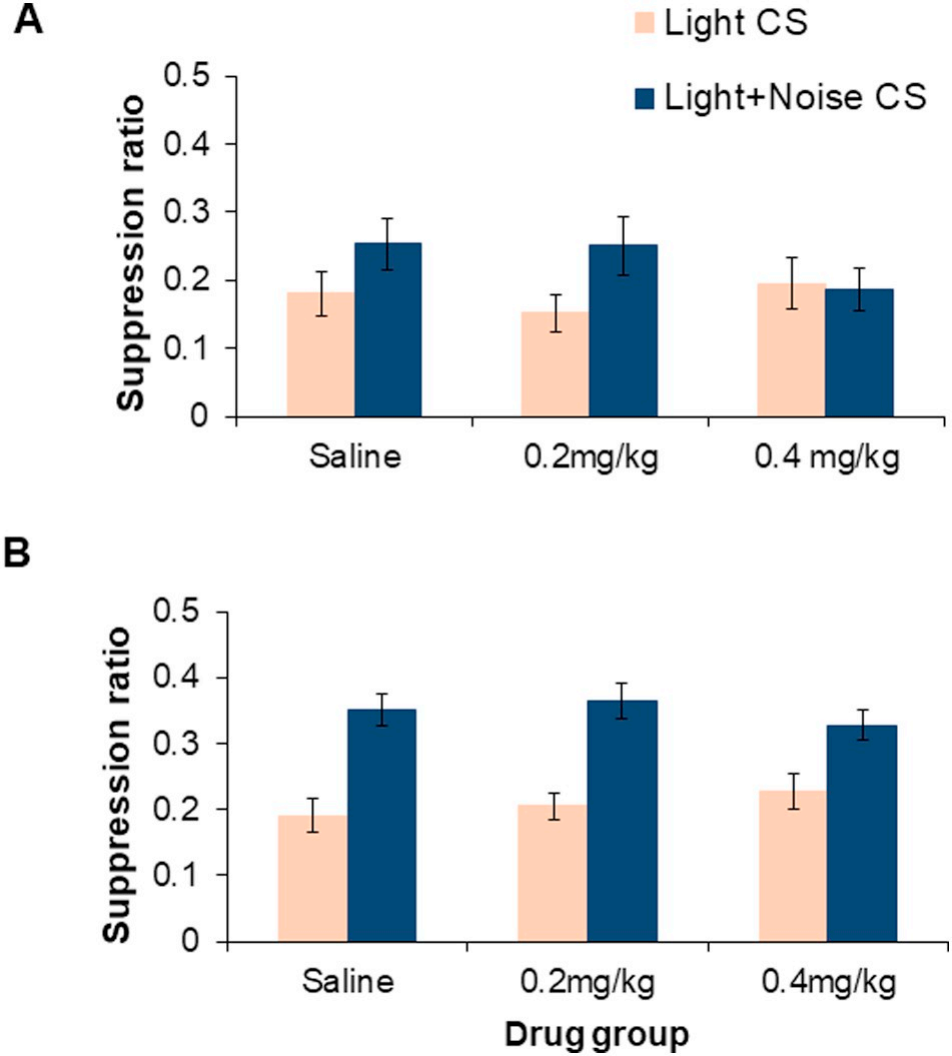
Experiment 2 light conditioned suppression test results shown as mean suppression ratio (±S.E.M.) subsequent to (A) one or (B) two conditioning sessions, for groups control (Light CS; light orange bars) and compound conditioned (Light + Noise CS; dark blue bars), following one (A) or two (B) treatments with saline, 0.2 or 0.4 mg/kg 8-OH-DPAT.

In both test sessions, prior to presentation of the CS, rats took the same amount of time overall to make 50 licks during the A period, by both drug and group condition [maximum *F*(2,66) = 0.765]. After one conditioning session only the effect of conditioning group approached significance for the suppression ratio measure of conditioning to the light [*F*(1,66) = 3.495, p = 0.066]. Fig. 2A shows that control rats conditioned to the light alone tended to be generally more suppressed than their overshadowed counterparts conditioned to the light compounded with the competing noise stimulus. By the second light test session, there was now a clear overall effect of conditioning group [*F*(1,66) = 50.521, p < 0.001], because - as would be expected - the control rats were more suppressed to the light than their overshadowed counterparts conditioned to the light plus noise compound (Fig. 2B). There were no drug or drug by group interaction effects [maximum *F*(2,66) = 1.010].

The noise suppression tests showed an overall effect of conditioning group at both the first [*F*(1,66) = 96.045, p < 0.001] and second test sessions [*F*(1,66) = 213.797, p < 0.001]. Control rats (conditioned to the light CS in the absence of the noise) showing little unconditioned suppression to the noise stimulus (Table 2). The drug by conditioning group interaction approached significance [*F*(2,66) = 2.649, p = 0.078] in the first noise test session. However, there were no drug or drug by group interaction effects [maximum *F*(2,66) = 1.651] at the second noise test session.

**Table 2:** Experiment 2 noise conditioned suppression test results shown as mean suppression ratio (±S.E.M.) subsequent to (A) one or (B) two conditioning sessions, for groups control (Light CS) and compound conditioned (Light + Noise CS), following one (A) or two (B) treatments with saline, 0.2 or 0.4 mg/kg 8-OH-DPAT. The Light CS group provides a measure of unconditioned suppression to the noise stimulus.

Drug/conditioning group	Saline	0.2 mg/kg 8-OH-DPAT	0.4 mg/kg 8-OH-DPAT
(A): Light CS	0.437 (±0.035)	0.424 (±0.021)	0.391 (±0.038)
(A): Light + Noise CS	0.158 (±0.046)	0.150(±0.043)	0.233 (±0.067)
(B): Light CS	0.507 (±0.033)	0.473 (±0.015)	0.450 (±0.023)
(B): Light + Noise CS	0.222 (±0.026)	0.184 (±0.012)	0.209 (±0.022)

## 4 Discussion

In line with earlier findings, there were no clear effects of 8-OH-DPAT on conditioning to the discrete cues provided by 5 s CSs, irrespective of the length of the trace interval used in Experiment 1, and irrespective of whether the CS took the form of a light alone, or a noise plus light compound in the Experiment 2 overshadowing procedure. Although the procedure was identical to that used previously with two conditioning trials (Nelson et al., 2011), in the present study the successful demonstration of overshadowing required the use of a second conditioning session to emerge. This allowed further evaluation of the effects of 8-OH-DPAT in that neither a weak nor a strong overshadowing effect was modulated by either drug treatment. The lack of effect on discrete cue conditioning is consistent with earlier reports contrasting effects of 8-OH-DPAT with those of other 5-HT compounds in other discrete cue conditioning procedures (Cassaday et al., 1993; Welsh et al., 1998; Inoue et al., 2011). However in the present study, treatment with 8-OH-DPAT was not completely ineffective in that competition from contextual cues was reduced in rats conditioned at the 30 s trace interval. Thus the lack of any effect on overshadowing or conditioning over a trace interval shows robust salience gating under 8-OH-DPAT after treatment with an effective dosage regime. Of course the results of the present study do not exclude a role in such cue competition procedures for the many 5-HT receptor subtypes which are not susceptible to the effects of treatment with 8-OH-DPAT.

In line with studies of fear conditioning measured in freezing procedures (Inoue et al., 2011), the results of the present study do suggest that competition from contextual cues is special. In an earlier study conducted with the same CER procedure, 5-HT depletion increased suppression to background cues (both those provided by the conditioning chambers and an experimental background stimulus) but had no effect on suppression to discrete cues, irrespective of trace condition and modality (Cassaday et al., 2001). In the present study, rats previously treated with 0.2 or 0.4 mg/kg 8-OH-DPAT were less suppressed to context, and this effect was seen in the 30 s trace groups when contextual conditioning was measured as the reshaping latencies (hesitancy to drink in the experimental chambers after conditioning, shown in Fig. 1A). The same pattern was reflected in suppression to the experimental background stimulus at 0.4 mg/kg 8-OH-DPAT (Fig. 1C). Thus, in line with prediction, contextual fear conditioning was reduced (Li et al., 2001, Li et al., 2006; Inoue et al., 2011). Earlier studies with the same CER procedure suggest that this latter effect was unlikely to relate to the stimulus modality of the experimental background (Cassaday et al., 2001; Norman and Cassaday, 2004) and in any event the cues provided by the conditioning chambers are multi-modal.

The substrates of discrete cue and contextual conditioning have been shown to be dissociated in studies of conventional lesions to the hippocampus (Hirsh, 1974; Phillips and Le Doux, 1992; Selden et al., 1991; Winocur et al., 1987). Moreover, a number of studies have shown that 5-HT_1A_ receptor agents modulate contextually conditioned fear (Homberg, 2012) and micro-injection studies have confirmed that hippocampus mediates the reduced contextual conditioning produced by 5-HT_1A_ agonists in freezing studies (Li et al., 2006). Since the level of conditioning supported by contextual cues is generally lower than that supported by discrete cues, one possibility is that such dissociations relate to baseline in that reduced conditioning after a lesion or drug treatment is easier to show when conditioning if not at ceiling (for whatever reason). The results of the present study are inconsistent with this interpretation in that the suppression ratios resulting from presentation of the light background stimulus were comparable to those resulting from presentation of the discrete cue previously presented at the 30 s trace interval, yet treatment with 8-OH-DPAT only affected conditioning to the former.

The fact that there was no similar effect of the same doses of 8-OH-DPAT on contextual cue competition in an overshadowing variant of the same CER procedure may also point to the importance of the serial aspect of competition from contextual cues in the trace procedure. The fact that there was no reduced suppression to context in rats conditioned at the 3 rather than the 30 s trace rather suggests that the interval over which contextual cues compete for associative strength is a determinant of 8-OH-DPAT effects thereon. In any event, although there was cue competition in both procedures, contextual conditioning in trace procedures seems to rely on different underlying mechanisms in that there was differential sensitivity to modulation by 8-OH-DPAT.

The actions of lower doses of 8-OH-DPAT, up to and including 0.2 mg/kg, are typically attributed to the activation of presynaptic 5-HT_1A_ autoreceptors (Warburton et al., 1997; Millan et al., 2008). Such an action results in a general reduction in 5-HT release (Sharp et al., 1989, Sharp et al., 1993). The actions of higher doses of 8-OH-DPAT, 0.4 mg/kg and above, may rather be due to the activation of postsynaptic 5-HT_1A_ receptors in projection areas such as hippocampus and frontal cortex (Araneda and Andrade, 1991; Misane et al., 1998; Stiedl et al., 2000; Zeeb et al., 2009). Thus, based on the doses in use, the present study does not allow any firm conclusion as to the relative contribution of pre- versus postsynaptic 5-HT_1A_ receptors. Moreover, a role for 5-HT_7_ receptors cannot be excluded and the profile of 5-HT receptor activation must be key in that - using the same overshadowing procedures - treatment with the selective serotonergic reuptake inhibitors setraline and fluvoxamine *increased* suppression to contextual cues (Cassaday and Thur, 2015).

In other preclinical studies, 8-OH-DPAT has been found to have anxiolytic properties (Li et al., 2001, Li et al., 2006). As in the present study, previous studies have similarly tended to focus on the acquisition rather than the expression of conditioned fear (but see e.g. Li et al., 2006), most have examined contextual conditioning using freezing procedures and few have examined discrete cue conditioning (Li et al., 2006; Inoue et al., 2011). To our knowledge, none have explicitly investigated cue competition in trace conditioning or overshadowing procedures. The results of the present study suggest that reduced competition from contextual cues at the point of conditioning may contribute to the anxiolytic properties of 8-OH-DPAT. Normally in a CER procedure, the introduction of a trace interval between CS and UCS has the consequence that suppression to contextual cues is increased, shown as longer latencies to drink (Fig. 1A). This effect was reversed under 0.4 mg/kg 8-OH-DPAT so that suppression to contextual cues was reduced in those rats conditioned with the longer (30 s) trace interval. Such a profile of action would be expected to reduce the capacity of intervening contextual cues to trigger fear responses.

It is a limitation of the present study that we only used male Wistar rats. Future studies should also include females to rectify this omission and test the generality of the findings. Stage of the oestrus cycle may result in behavioural variability in rodents, particularly in aversively-motivated procedures, but behavioural variability in males may be just as great, for example when they are group housed or otherwise exposed to odour of other males (Prendergast et al., 2014).
